# Skull base osteomyelitis: factors implicating clinical outcome

**DOI:** 10.1007/s13760-019-01110-w

**Published:** 2019-03-06

**Authors:** Jacek Sokołowski, Magdalena Lachowska, Emilia Karchier, Robert Bartoszewicz, Kazimierz Niemczyk

**Affiliations:** Department of Otolaryngology, Medical University of Warsaw, Warsaw, Poland

**Keywords:** Skull base, Osteomyelitis, Otitis media, Cranial nerve

## Abstract

Skull base osteomyelitis is a serious disease with a high risk of complications including neuroinfection. Typically, the inflammation of the skull base results from infection from neighboring tissues. In case of malignant otitis externa, inflammation disseminates from the external auditory canal. In this study, we present our experience with seven patients diagnosed with skull base osteomyelitis that began with otitis externa and have been treated in our department for the last 10 years. Department Patient Database was searched for the diagnosis skull base osteomyelitis. The search covered the last 10 years. The search revealed seven patients who met the above-described criteria. Medical records of those patients were carefully analyzed including age, gender, symptoms and signs, diagnostics details, treatment, performed procedures, number of hospitalization days, comorbid diseases, and complications including any cranial nerve palsy. Detailed analysis of medical records of patients included in this study showed that skull base osteomyelitis presents a challenge for diagnosis and treatment. Treatment strategy requires prolonged aggressive intravenous antibiotic therapy, and in some cases combined with surgical intervention. Cranial nerve paresis indicates progression of the disease and is associated with longer hospital stay. Similar relationship is observed in patients with skull base osteomyelitis that required surgery. Diabetes in patient’s medical history may complicate the healing process. Diabetes, neural involvement, and surgery may overlap each other resulting in longer hospital stay. Cranial nerve paresis may not resolve completely and some neural deficits become persistent.

## Introduction

Skull base osteomyelitis is a serious disease with a high risk of complications including neuroinfection. Typically, the inflammation of the skull base results from infection from neighboring tissues. It is a complication of ear or paranasal sinuses’ infection mostly seen in immunocompromised patients older than 65 years of age, especially with diabetes, hematological disorders (e.g., leukemia or granulocytopaenia) or arteriosclerosis [1–3]. At the beginning, a course of the disease is often polysymptomatic which may delay its diagnosis. In case of malignant otitis externa, inflammation disseminates from the external auditory canal (EAC) to the cartilages via the Santorini fissures and the tympanomastoid suture [1, 2]. Skin infection of the EAC quickly progresses into osteitis of the temporal bone due to the lack of any subcutaneous tissue in the EAC. The infection of the temporal bone continues to spread further rapidly through the combination of air cells and Haversian nutrient channels. Involvement of the stylomastoid foramen can lead to the facial nerve palsy, and in addition, the jugular foramen inflammation results in paralysis of the lower group of the cranial nerves (CN) [4–8].

Initially, the dominant symptom is otalgia, headache, and often also discharge from the ear accompanied by conductive hearing loss often resulting from a swelling of the EAC or formation of granulation tissue [7–10]. Usually, the symptoms are treated locally for some time, without extensive diagnostics. This leads to the serious life-threatening condition with neurological symptoms and the patient is admitted to the hospital. At that time, the computed tomography (CT) may reveal the fluid filling the mastoid air cells and magnetic resonance imagining (MRI) shows the inflammation of the skull base tissues. However, imaging scans present poor diagnostic value in the diagnosis of bone inflammation [2]. A reliable confirmation test is scintigraphy with titanium and indium-labeled leukocytes.

Treatment of skull base inflammation due to the malignant otitis externa involves prolonged administration of antibiotics and debridement of the infected tissues, and in some cases, antifungal medication is required [11].

The prognosis in skull base osteomyelitis is serious with the possibility of recurrence, which results in subsequent hospitalizations and high mortality rate [12, 13]. CN paresis always indicates serious complication and is often associated with deterioration of the prognosis for recovery [4, 11, 14, 15].

In this study, we present our experience with seven patients diagnosed with skull base osteomyelitis that began with otitis externa and have been treated in our department for the last 10 years.

## Methods

Department Patient Database was searched for the diagnosis of “malignant otitis externa”, “skull base osteomyelitis”, “otitis externa”, “skull base tumor”, and “otitis externa” that required hospitalization, “skull base inflammation”, and “sigmoid sinus thrombophlebitis”. The search covered the last 10 years. The search revealed seven patients who met the above-described criteria. Medical records of those patients were carefully analyzed including age, gender, symptoms and signs, diagnostics details, treatment, performed procedures, number of hospitalization days, comorbid diseases, and complications including any CN palsy.

The analyses were conducted with the Excel software (Microsoft Corp. Redmond, WA, USA).

The study conforms to The Code of Ethics of the World Medical Association (Declaration of Helsinki). This is a retrospective study and no free informed consent form was needed, and subject’s identity was not divulged. The study was approved by the local Institutional Ethics Committee Review Board, where the study was conducted.

## Results

### General patients’ characteristics

Diagnosis of “skull base inflammation” occurred in 28.57% of cases in the group as the “initial diagnosis” and in 85.71% as the “final diagnosis”. Three patients were male (42.86%) and four were female (57.14%). The median age was 61 years (average 63, min. 32, max. 83), and two patients were over 80 years. In all patients, the skull base inflammation was localized on one side, in 71.43% on the left sand in 28.57% on the right. Tables 1 and 2 summarize clinical characteristics of all seven patients analyzed in this study.

**Table 1:** Clinical characteristics of each patient diagnosed with skull base osteomyelitis: gender, age, comorbid diseases, initial and final diagnosis, side of infection, and hospitalization time

Patient #	Gender	Age	Comorbid diseases	Initial diagnosis	Final diagnosis	Side of infection	Hospitalization time
1	F	59	Diabetes Adison–Biermer anemia Allergic rhinitis	Otitis externa	Malignant otitis externa. Skull base inflammation	L	92
2	M	60	Diabetes	Petrous apicitis	Petrous apex abscess. Skull base inflammation	R	75
3	F	65	Diabetes	Skull base inflammation	Skull base inflammation	R	16
4	M	81	–	Skull base inflammation	Skull base inflammation	L	59
5	F	61	–	Chronic otitis media	Skull base inflammation	L	3
6	M	83	–	Malignant otitis externa	Malignant otitis externa	L	12
7	F	32	Diabetes	Chronic otitis media	Chronic otitis media. Skull base inflammation	L	8

**Table 2:** Clinical findings on admission, complications, microbiology results, and treatment details of each patient diagnosed with skull base osteomyelitis

Patient #	Clinical findings on admission	Complications	Culture	Combined parenteral antibiotic therapy	Performed surgery
1	Otalgia, severe headache, polyp in EAC, swollen EAC, TM perforation, otorrhea	Facial nerve paresis meningitis, sepsis	*Pseudomonas aeruginosa*	Ciprofloxacin, ceftriaxone, clindamycin	Removal of the polyp from the external auditory canal, decompression of the facial nerve, antromastoidectomy, tympanoplasty
2	Severe headache, otorrhea, TM perforation	Facial nerve paresis	Pseudomonas, * Aspergillus flavus*	Vancomycin, itraconazole, ciprofloxacin	Radical mastoidectomy
3	Severe otalgia	–	Negative	Ciprofloxacin	–
4	Headache, hoarseness, vocal fold palsy, soft palate paresis, otalgia	Vagal nerve and glossopharyngeal nerve paresis	Negative	Ceftriaxone, metronidazole	Parapharyngeal space drainage
5	Swollen mastoid	–	Negative	clindamycin, amoxicillin and clavulanate acid	–
6	Otalgia, swollen EAC, swollen mastoid, TM perforation, otorrhea	–	Negative	Amoxicillin and clavulanate acid, metronidazole	Antromastoidectomy, tympanoplasty
7	Otalgia, reddened TM, swollen mastoid	–	Negative	Ciprofloxacin	–

*EAC* external auditory canal, *TM* tympanic membrane

### Comorbid diseases

The most common comorbid illness was diabetes observed in 57.14% of analyzed patients. In case of diabetes as the comorbid disease, the hospitalization time was longer with median of 45.5 days (average 47.75 days) than in case of patients without diabetes in their history (median 12 days, average 24.67 days). One patient suffered from Addison-Biermer disease and allergic rhinitis in addition to diabetes and her hospitalization time was the longest, 92 days.

### Clinical findings and complications

In six patients (85.71%), the analysis revealed disorders within temporal bone of which four presented more than one. The temporal bone disorders were as follows: severe otalgia, otorrhea, polyp of the external ear canal, tympanic membrane perforation, mastoid process swelling, swelling of the external auditory canal, and reddened tympanic membrane.

In three cases, CN paresis developed, facial in two patients, and vagal (recurrent laryngeal nerve) and glossopharyngeal nerve in one. In one patient, after facial nerve involvement meningitis developed. Two patients with cranial nerve paresis required prolonged narcotic analgesics therapy. In these two cases, severe pain of the entire head occurred 18 and 26 days before CN paresis which was their cause to refer to hospital seeking help.

### Imaging scans

**Fig. 1:**
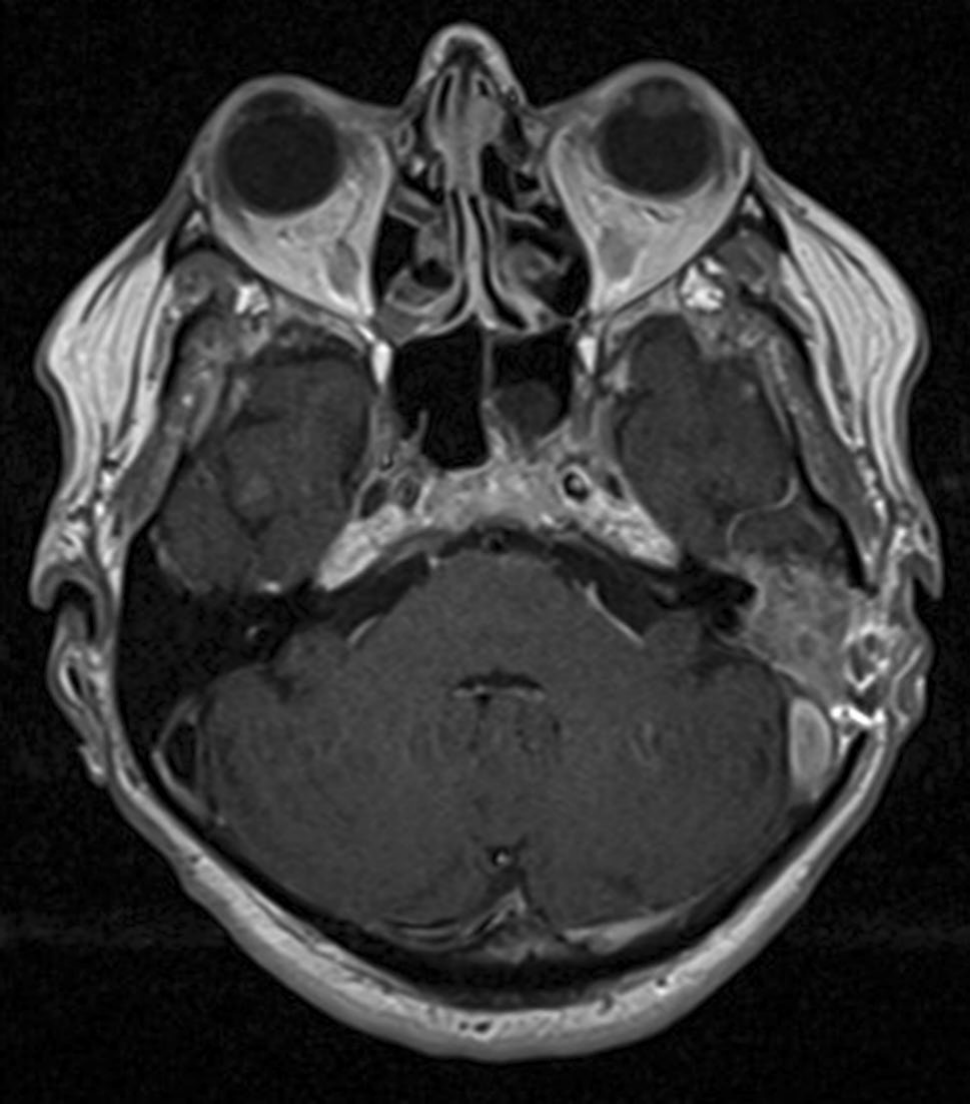
Axial T1-weighted MR images demonstrating an enhancing soft-tissue formation in the mastoid process and thrombosis of the sigmoid sinus

CT scans revealed increased soft tissue in the tympanic cavity along with bony destruction of the mastoid in one patient. In two patients, MR showed soft-tissue abnormalities over the mastoid area and in addition in one, sigmoid sinus thrombophlebitis (Fig. 1). In one case, the MR images revealed enhancing soft-tissue formation in the parapharyngeal space.

### Pathogens and treatment

The administered treatment included long-term intravenous broad-spectrum antibiotic therapy as follows using ciprofloxacin, ceftriaxone combined with metronidazole. Less frequently the following were used: clindamycin, amoxicillin and clavulanate acid, vancomycin, and itraconazole. Targeted broad-spectrum antibiotic therapy was used in two patients. In those two cases, culture revealed *Pseudomonas aeruginosa*, including one combined with Aspergillus. In other five cases, culture result was negative.

Three patients were treated only with antibiotics. In the other four, in addition to antibiotics surgery was performed. In those cases, the median time elapsed from admission to the hospital to the surgery was 11.5 days (average 13.25 days). The most frequently performed surgery was antromastoidectomy (3 cases) with one patient undergoing radical mastoidectomy.

In one patient, who developed facial nerve paresis followed by meningitis, facial nerve decompression was performed along with antromastoidectomy and tympanoplasty. After this treatment combination, her condition improved.

One patient required parapharyngeal space drainage.

### Hospitalization time

The presence of CN paresis significantly affected the duration of hospital stay resulting in the median hospitalization time of 75 days (average of 75.33 days). In contrast, the hospital stay in case of patients without CN paresis was shorter, with median duration of hospital stay 10 days (average 9.75 days). When it comes to diabetes, two patients out of four with diabetes had to stay longer in hospital. In cases that required surgery, hospitalization time was significantly longer with median duration of 67 days (average of 59.5 days). Without surgery, median hospitalization time was 8 days (average of 9 days).

In one case, after cranial nerve involvement meningitis developed and this patient was the one with the longest hospital stay of 92 days.

### Prognosis and survival

None of the analyzed patients died. All of them were dismissed form hospital in a good condition; however, in described three cases with neural paresis, the problems reduced with time but persisted in two of them.

## Discussion

Skull base osteomyelitis is a complex clinical entity and remains a great challenge due to rarity of the condition, however, with increasing prevalence, not fully analyzed prognostic factors and high mortality rate [3–5, 8, 11, 12, 15, 16]. Diagnosis of the disease is difficult, because there is no single pathognomonic criterion that defines malignant otitis externa and skull base inflammation. It is based on clinical, laboratory, and radiological findings. In this paper, we discuss SBO in the context of otitis externa.

### Age and comorbid diseases

The skull base inflammations described in the literature most often affect diabetic or immunodeficient patients [2, 17–19]. Diabetics are more prone to skull base osteomyelitis [3, 8–10, 17, 19, 20]. In our study, the diabetes was observed in 57.14% of cases, in addition, one patient suffered from Adison–Biermer anemia and diabetes.

SBO is also predominantly seen in older patients [8, 10, 15, 17, 21]. In our study, most of the patients were over 60 years. One patient in our study was very young aged 32 years. In the literature, acquired immune deficiency syndrome (AIDS) patients who develop malignant external otitis tend to be younger than the typical patient with this ear infection, and most of them are not diabetic [2, 10, 16]. In our study, there was no patient diagnosed with AIDS including the youngest one; however, he had diabetes running in his history.

### Clinical findings, cranial nerves involvement and other complications

Frequent symptom in malignant otitis externa is severe otalgia accompanied by purulent otorrhea [8, 10, 11, 15, 17, 21] found in three patients (42.86%); however, otitis and/or otorrhea was found in 85.71% of analyzed cases in this study. On physical examination, the typical finding is granulation tissue/polyp located in the EAC at the level of bony–cartilaginous junction [15, 17, 21] that was found in one patient. In our study, the pathologic changes in the EAC were found in four cases with perforation of the tympanic membrane in three of them. On admission, patients were without fever, and the number of white blood cells was within normal range.

Previous studies have described facial nerve dysfunction and other CN involvement in skull base osteomyelitis. The occurrence of CN paresis indicates progression of the disease [4, 8, 11, 14, 15, 21]. Furthermore, patients with any CN involvement experience usually worse outcomes; in some of them, the neurological deficits do not resolve completely [4, 8, 18, 21, 22]. This was observed in two of the three patients with CN paresis analyzed in this study. However, in the literature, there are also cases described with completely restored CN functions after their initial paresis due to SBO [15, 18]. One of our patients with neural paresis displayed complete recovery.

It should be emphasized that inadequate severe headache involving the whole head compared to the mild changes observed in the otoscopy may occur quite long before the CN paresis [21, 23] which was also described in medical records of our two patients. In those cases, the pain started 18 and 26 days before facial nerve paresis.

### Imaging techniques

MRI or CT as single modality is insufficient to be crucial in diagnosing malignant otitis externa [24]. Technetium 99m scans may show osteoblastic activity that may be useful for therapeutic monitoring of SBO [25]. Hybrid technique PET–MRI or CT-MRI present complementary value that strengthens imaging modalities [24, 26]. However, soft-tissue abnormalities in MRI over the mastoid area and/or in infratemporal fossa, or CT scan demonstrating the presence of bony destruction in mastoid area, and clinical manifestations of otitis allow initial diagnosis of malignant otitis externa [4, 8, 15, 21, 22, 24, 25]. In our study, CT scans revealed inflammatory process in the tympanic cavity with features of bony destruction of the mastoid process in one patient. MR images showed soft-tissue abnormalities in three patients.

### Pathogens and antibiotic treatment

In case of the skull base osteomyelitis, treatment is based primarily on aggressive broad-spectrum intravenous antibiotic therapy lasting 6–20 weeks [7, 8, 11, 15, 18, 19]. The inflammatory process can be caused by any pathogen; however, in most cases, *P. aeruginosa* is found in culture [4, 8–11, 15, 17]. In the treatment, a combination therapy of different antibiotics is used: fluoroquinolones, third-generation cephalosporins, aminoglycosides, carbapenems, and vancomycin are used [7, 11, 15, 18, 19, 27]. In some cases, antifungal medication is required [11], which was the case in one of our patients. In our study, quinolones were the most commonly used antibiotics, although less frequently than described in the literature. In 71.43% of our patients, the culture was negative and their antibiotic treatment was carried out empirically. The possible case was that antibiotic treatment had stared before the specimens were taken to the culture which resulted in its negative result.

### Surgical treatment

Cleaning the EAC from an inflammatory debris is one of the recognized methods of malignant otitis externa treatment [7, 18]. In case of intracranial complications, surgical intervention may be indispensable and various procedures are used depending on the severity of the inflammatory process, e.g., facial nerve decompression, antromastoidectomy, radical mastoid surgery, or even drainage of the skull base [1, 4, 11, 15, 19]. Our experience shows that inflammation can also be significantly reduced with surgical treatment. In those cases, the surgery is performed with the intend to remove the source of the infection. Therefore, in our department, we use combined therapy of intravenous prolonged antibiotics administration and surgical treatment in some SBO patients. This combined treatment was performed in 57.14% of analyzed subjects and achieved a positive final outcome in all of them. There was one patient in our study that displayed vocal fold palsy and glossopharyngeal nerve paresis manifested with hoarseness, dysarthria and dysphagia. The parapharyngeal space drainage was performed with addition to antibiotic administration.

### Hospitalization time

In the literature, in patients with SBO hospitalization, time is usually longer in cases of comorbidities and/or CN paresis [3, 8, 11, 21]. Our study stays in line with those statements, the presence of neural paresis significantly prolonged duration of hospital stay with median time being 10.71 weeks in contrast to 1.43 weeks in patients without neural deficits. When it comes to diabetes, the analysis did not show obvious connection affecting time frames. Half of our patients with diabetes had to stay longer in hospital (13.14 and 10.71 weeks), and the other half did not (2.29 and 1.14 weeks).

Patients, who receive surgery with therapeutic intent, usually require longer hospital stay [18]; however, removing infectious tissues and decreasing inflammatory load may help to improve antibiotics penetration to the region and, therefore, in some SBO cases is required. In this study, the patients who underwent surgery stay significantly longer in hospital (median 9.57 weeks) than those without surgical intervention (median 1.14 weeks). However, all the three patients with neural involvement required surgery and it complicates analysis of hospitalization time, because in those three cases, two factors overlapped each other and both might affect the time outcome with unknown superiority of one of them. In addition, two out of those mentioned three patients had diabetes that also overlapped the time of hospitalization and blured the picture.

### Survival rate

SBO is a serious clinical condition with overall mortality rate reaching 10% [18, 21]. In our study, none of the patients died including the ones with CN involvement. It stays in line with some other authors [4, 11]. This may be associated with introduction of new antibiotics and advances in surgical procedures in the last years.

## Conclusions

Skull base osteomyelitis presents a challenge for diagnosis and treatment. Treatment strategy requires prolonged aggressive intravenous antibiotic therapy, and in some cases combined with surgical intervention. Cranial nerve paresis indicates progression of the disease and is associated with longer hospital stay. Similar relationship is observed in patients with SBO that require surgery. Diabetes in patient’s medical history may complicate the healing process. Diabetes, neural involvement, and surgery may overlap each other resulting in longer hospital stay. Cranial nerve paresis may not resolve completely and some neural deficits become persistent.
